# Supplementary material for: Comparative genomics provides new insights into the diversity, physiology, and sexuality of the only industrially exploited tremellomycete: Phaffia rhodozyma
Source: BMC Genomics. 2016 Nov 9;17:901. doi: 10.1186/s12864-016-3244-7 (PMC5103461; doi:10.1186/s12864-016-3244-7)
Supplement: Additional file 6: — List of orphan genes with links to PFAM (related to Additional file 1: Table S1). (ZIP 1428 kb) [file 12864_2016_3244_MOESM6_ESM.zip › PFAM_UNIQUE/G01736_P.html]

#                                                                            --- full sequence --- -------------- this domain -------------   hmm coord   ali coord   env coord
  
# target name        accession   tlen query name           accession   qlen   E-value  score  bias   #  of  c-Evalue  i-Evalue  score  bias  from    to  from    to  from    to  acc description of target
  
#------------------- ---------- ----- -------------------- ---------- ----- --------- ------ ----- --- --- --------- --------- ------ ----- ----- ----- ----- ----- ----- ----- ---- ---------------------
  
G01736\_P             -            422 ORC6                 PF05460.8    353       3.6    5.3   8.0   1   2   0.00096      0.26    9.0   3.2    79   187    19   142    16   166 0.69 - Origin recognition complex subunit 6 (ORC6)  
G01736\_P             -            422 ORC6                 PF05460.8    353       3.6    5.3   8.0   2   2       3.9   1.1e+03   -2.9   0.2   115   139   197   215   170   285 0.49 - Origin recognition complex subunit 6 (ORC6)
